# Upregulation of miR-589-3p Contributes to Lung Adenocarcinoma Progression Through Inhibition of WWC2

**DOI:** 10.3390/cancers18091349

**Published:** 2026-04-23

**Authors:** Sultan F. Kadasah

**Affiliations:** Department of Biology, Faculty of Science, University of Bisha, P.O. Box 551, Bisha 61922, Saudi Arabia; sukadasah@ub.edu.sa

**Keywords:** lung adenocarcinoma, miR-589-3p, WWC2 protein, apoptosis, cell proliferation, oncogenes, tumor suppressor proteins

## Abstract

Lung adenocarcinoma (LUAD) is the most common subtype of non-small cell lung cancer and remains a leading cause of cancer-related mortality worldwide. MicroRNAs (miRNAs) are critical regulators of tumor progression; however, the biological role and molecular mechanisms of miR-589-3p in LUAD remain unclear. In this study, the expression levels of miR-589-3p and WWC2 were analyzed using The Cancer Genome Atlas lung adenocarcinoma (TCGA-LUAD) datasets via the UALCAN platform. Flow cytometric apoptosis analysis and functional assays including CCK-8, colony formation, AO/EB staining, and Transwell invasion assays were performed in LUAD cell lines. The interaction between miR-589-3p and WWC2 was validated using dual-luciferase reporter assays, Western blotting, and rescue experiments. miR-589-3p expression was significantly elevated in LUAD tissues compared with normal lung tissues (*p* < 0.05) and was positively associated with an advanced tumor stage and lymph node metastasis (*p* < 0.05). Inhibition of miR-589-3p significantly suppressed proliferation and colony formation (*p* < 0.05), reduced invasive capacity (*p* < 0.05), and markedly increased apoptosis (*p* < 0.01) in LUAD cells. Dual-luciferase reporter assays confirmed WWC2 as a direct target of miR-589-3p, with miR-589-3p mimics significantly reducing WWC2 wild-type reporter activity (*p* < 0.05). WWC2 expression was significantly downregulated in LUAD tissues (*p* < 0.05), and WWC2 knockdown reversed the anti-proliferative, pro-apoptotic, and anti-invasive effects induced by miR-589-3p inhibition (*p* < 0.01). These findings demonstrate that miR-589-3p promotes lung adenocarcinoma progression by directly suppressing WWC2. The miR-589-3p/WWC2 axis represents a novel molecular mechanism contributing to LUAD malignancy and may provide a foundation for future mechanistic and translational studies.

## Simple Summary

Lung adenocarcinoma (LUAD) is a major cause of cancer-related deaths worldwide. This study found that miR-589-3p is highly expressed in LUAD and is associated with tumor progression. Functional experiments showed that miR-589-3p promotes cancer cell growth and invasion while reducing apoptosis. Mechanistically, miR-589-3p drives LUAD progression by directly targeting and suppressing the tumor suppressor gene WWC2.

## 1. Introduction

Lung cancer remains the leading cause of cancer-related mortality worldwide and continues to pose a substantial public health burden despite advances in diagnosis and treatment strategies [1,2,3]. Among its histological subtypes, lung adenocarcinoma (LUAD) represents the most prevalent form of non-small cell lung cancer (NSCLC) and accounts for a significant proportion of lung cancer-associated deaths [4,5]. Although recent developments in targeted therapies and immunotherapy have improved outcomes in selected patient populations, the overall survival for patients with LUAD remains unsatisfactory, largely due to tumor heterogeneity, therapeutic resistance, and metastatic progression [6,7,8]. These challenges highlight the need for a deeper understanding of the molecular mechanisms underlying LUAD initiation and progression. MicroRNAs (miRNAs) are a class of endogenous, small non-coding RNAs that regulate gene expression at the post-transcriptional level by binding to complementary sequences in the 3′-untranslated regions (3′-UTRs) of target mRNAs, leading to mRNA degradation or translational repression [9,10]. Accumulating evidence indicates that miRNAs play critical roles in cancer-related biological processes, including cell proliferation, apoptosis, invasion, metastasis, and therapy resistance [9,10,11,12]. In lung adenocarcinoma, dysregulation of multiple miRNAs has been shown to contribute to tumor growth and malignant behavior, highlighting the importance of miRNA-mediated regulatory networks in LUAD pathogenesis [9]. However, despite extensive research, a large number of miRNAs remain functionally uncharacterized in LUAD.

MicroRNA-589 (miR-589) has emerged as a context-dependent regulator in several malignancies, exhibiting either tumor-suppressive or oncogenic properties depending on the cancer type and cellular context. In breast cancer, miR-589 has been reported to function as a tumor suppressor by directly targeting metastasis-associated protein 2 and inhibiting tumor progression [13]. Conversely, oncogenic roles of miR-589 have been described in gastric cancer, pancreatic cancer, and hepatocellular carcinoma, where miR-589 promotes proliferation, invasion, stemness, and chemoresistance through diverse signaling pathways [14,15,16,17,18,19,20]. Notably, epigenetic regulation of the miR-589 promoter has also been linked to malignant progression in NSCLC, suggesting a potential role for this miRNA family in lung cancer biology [12]. Nevertheless, the expression pattern, functional significance, and downstream molecular targets of miR-589-3p in lung adenocarcinoma remain largely unexplored.

WWC2 (WW and C2 domain-containing protein 2) is a member of the WWC protein family and has been implicated in the regulation of cell polarity, proliferation, and invasion, partly through its involvement in Hippo signaling and related tumor-suppressive pathways [21,22,23,24]. Increasing evidence suggests that WWC2 acts as a tumor suppressor in multiple cancer types, including hepatocellular carcinoma and colorectal cancer, where reduced WWC2 expression is associated with enhanced invasion and metastatic potential [22,23,24]. In lung adenocarcinoma, WWC2 has been identified as a functional target of oncogenic miRNAs, and its downregulation has been linked to increased proliferation and invasion of LUAD cells [25]. However, whether WWC2 is regulated by miR-589-3p in LUAD has not been investigated.

In the present study, we systematically investigated the expression and biological function of miR-589-3p in lung adenocarcinoma using integrated bioinformatics analysis of TCGA-LUAD datasets and in vitro experimental validation. We further explored the regulatory relationship between miR-589-3p and WWC2 and elucidated the role of the miR-589-3p/WWC2 axis in LUAD cell proliferation, apoptosis, and invasion.

## 2. Materials and Methods

### 2.1. Bioinformatics Analysis of TCGA-LUAD Datasets

The expression profiles of miR-589-3p and WWC2 in lung adenocarcinoma (LUAD) were analyzed using data derived from The Cancer Genome Atlas (TCGA). The UALCAN online platform (https://ualcan.path.uab.edu) [26,27] was used to evaluate differential expression between normal lung tissues and primary LUAD tissues and to perform subgroup analyses based on patient sex, tumor stage, and nodal metastasis status. TCGA miRNA-seq and mRNA-seq datasets were analyzed independently, and differences in sample size reflect data availability for each dataset. Statistical significance was calculated automatically by the UALCAN platform using Student’s *t*-test, with *p* < 0.05 considered to indicate statistical significance.

### 2.2. Cell Culture

The human lung adenocarcinoma cell lines A549, H1975, PC9, and H1299, as well as normal human bronchial epithelial (HBE) cells, were obtained from the Cell Bank of the Chinese Academy of Sciences (Shanghai, China). Cells were cultured in Dulbecco’s Modified Eagle Medium (DMEM; Gibco, Thermo Fisher Scientific, Waltham, MA, USA) supplemented with 10% fetal bovine serum (FBS; Gibco), 100 U/mL penicillin, and 100 μg/mL streptomycin (Gibco). All cells were maintained at 37 °C in a humidified incubator containing 5% CO_2_.

### 2.3. Cell Transfection

miR-589-3p inhibitor, inhibitor negative control (inhibitor-NC), miR-589-3p mimics, small interfering RNA targeting WWC2 (si-WWC2), and corresponding negative controls were synthesized by GenePharma (Shanghai, China). The mature sequence of hsa-miR-589-3p was 5′-UCAGAACAAAUGCCGGUUCCCAGA-3′. The miR-589-3p mimic sequences were as follows: sense, 5′-UCAGAACAAAUGCCGGUUCCCAGA-3′; antisense, 5′-UGGGAACCGGCAUUUGUUCUGAUU-3′. The miR-589-3p inhibitor sequence was 5′-UCUGGGAACCGGCAUUUGUUCUGA-3′, and the inhibitor negative control sequence was 5′-CAGUACUUUUGUGUAGUACAA-3′. The si-WWC2 sequences were as follows: sense, 5′-GGGAAGCAGGGTTTGACCCTC-3′; antisense, 5′-GCTTCACTGAGTTCATACCCGC-3′. The pcDNA3.1-WWC2 overexpression plasmid and empty vector control were obtained from GeneChem (Shanghai, China). Cell transfections were performed using Lipofectamine 3000 reagent (Invitrogen, Thermo Fisher Scientific, USA) according to the manufacturer’s instructions. The final concentrations used for transfection were 2.5 µg/well for pcDNA3.1 constructs and 50 nM for miRNA mimics, inhibitors, and siRNAs.

### 2.4. Quantitative Real-Time PCR (qRT-PCR)

Total RNA was extracted using TRIzol reagent (Invitrogen, Carlsbad, CA, USA). For miRNA detection, reverse transcription was performed using a miRNA First Strand cDNA Synthesis Kit (Takara, Shiga, Japan), while mRNA was reverse-transcribed using a PrimeScript RT reagent Kit (Takara). qRT-PCR was conducted using SYBR Green Master Mix (Takara) on a QuantStudio™ 6 Flex Real-Time PCR System (Applied Biosystems, Carlsbad, CA, USA). U6 small nuclear RNA was used as an internal control for miR-589-3p, and GAPDH was used for WWC2 mRNA normalization. Relative expression levels were calculated using the 2^−ΔΔCt^ method. The primers used in this study are presented in Supplementary Table S1.

### 2.5. Cell Proliferation Assay

Cell proliferation was assessed using the Cell Counting Kit-8 (CCK-8; Dojindo Laboratories, Tabaru, Japan). Transfected A549 and H1299 cells were seeded into 96-well plates at a density of 2 × 10^3^ cells per well. At 0, 24, 48, 72, and 96 h, 10 μL of CCK-8 solution was added to each well and incubated for 2 h at 37 °C. Absorbance was measured at 450 nm using a microplate reader (Bio-Rad, Hercules, CA, USA).

### 2.6. Colony Formation Assay

Transfected cells were seeded into 6-well plates at a density of 500 cells per well and cultured for 10–14 days. Colonies were fixed with 4% paraformaldehyde and stained with 0.1% crystal violet solution. Colonies containing more than 50 cells were counted under a light microscope. The colony formation results were quantified and expressed as a percentage relative to the total number of colonies observed in the control group.

### 2.7. Target Gene Prediction and Luciferase Reporter Assay

Potential target genes of miR-589-3p were predicted using the TargetScan, miRDB, and OncomiR databases, and the top 200 targets were selected from each database. These targets were selected to balance sensitivity and specificity, allowing the inclusion of high-confidence candidates while minimizing false positives. The wild-type (WT) or mutant (MUT) 3′-UTR sequences of WWC2 were cloned into the multiple cloning site of the pmirGLO vector (Promega, Madison, WI, USA) between the SacI and XbaI restriction sites. A549 cells were co-transfected with reporter constructs and miR-589-3p mimics or miR-NC using Lipofectamine 3000. After 48 h, luciferase activity was measured using a Dual-Luciferase Reporter Assay System (Promega) according to the manufacturer’s instructions.

### 2.8. Western Blot Analysis

Total protein was extracted using RIPA lysis buffer containing protease inhibitors (Beyotime, Shanghai, China). The protein concentration was determined using a BCA Protein Assay Kit (Thermo Fisher Scientific). Equal amounts of protein were separated via SDS-PAGE and transferred onto PVDF membranes (Millipore, Burlington, MA, USA). Membranes were blocked with 5% non-fat milk and incubated overnight at 4 °C with primary antibodies against WWC2 (Santa Cruz Biotechnology, Dallas, TX, USA, Cat# sc-515892, mouse monoclonal, 1:1000), Bax (Cell Signaling Technology, Danvers, MA, USA, Cat# 2772, rabbit monoclonal, 1:1000), Bcl-2 (Cell Signaling Technology, USA, Cat# 3498, rabbit monoclonal, 1:1000), and β-actin (Cell Signaling Technology, USA, Cat# 4970, rabbit monoclonal, 1:2000). After incubation with HRP-conjugated secondary antibodies (anti-rabbit or anti-mouse IgG; Abcam, Cambridge, UK, 1:5000), protein bands were visualized using enhanced chemiluminescence reagent (ECL; Thermo Fisher Scientific). β-actin was used as the internal loading control. All comparative samples were run on the same gel and processed under identical conditions. All Western blot experiments were performed with at least three independent biological replicates. A densitometric analysis of protein band intensity was performed using ImageJ software v1.53 (NIH, Bethesda, MD, USA). Band intensities were normalized to those of β-actin and expressed relative to those for the control groups.

### 2.9. AO/EB Staining Assay

Apoptosis was evaluated using acridine orange/ethidium bromide (AO/EB) staining (Solarbio, Beijing, China). Transfected cells were stained with AO/EB working solution and immediately observed under a fluorescence microscope (Olympus, Tokyo, Japan). Live cells fluoresced green, whereas apoptotic cells exhibited orange or red fluorescence.

### 2.10. Flow Cytometric Analysis of Apoptosis

Cell apoptosis was further quantified using the Annexin V-FITC/PI Apoptosis Detection Kit (BD Biosciences, San Jose, CA, USA). Transfected cells were harvested, washed, and stained according to the manufacturer’s instructions. Apoptotic cells were analyzed using a flow cytometer (FACSCanto II; BD Biosciences). Early and late apoptotic populations were quantified using FlowJo software Version 10.6.

### 2.11. Transwell Invasion Assay

Cell invasion was assessed using Transwell chambers with Matrigel-coated membranes (Corning, New York, NY, USA). Transfected cells were seeded into the upper chamber in serum-free medium, while medium containing 10% FBS was added to the lower chamber. After 24 h, invaded cells were fixed, stained with crystal violet, and counted under a microscope.

### 2.12. Statistical Analysis

All experiments were performed in triplicate, and data are presented as the mean ± standard deviation (SD). Statistical analyses were conducted using GraphPad Prism 8.0 (GraphPad Software, San Diego, CA, USA). Comparisons between two groups were performed using Student’s *t*-test. Comparisons among multiple groups at a single time point were analyzed using one-way ANOVA followed by Tukey’s post hoc test. For time-course experiments, two-way repeated-measures ANOVA (with time and treatment as factors) was applied, followed by appropriate multiple-comparisons post hoc tests. A *p* value < 0.05 was considered statistically significant.

## 3. Results

### 3.1. miR-589-3p Is Significantly Upregulated in LUAD and Associated with Clinicopathological Characteristics

**Figure 1:**
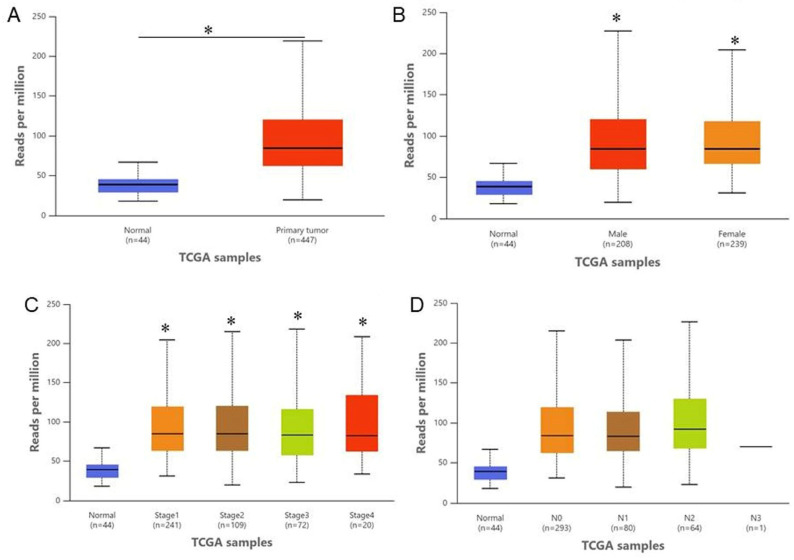
miR-589-3p is upregulated in LUAD and associated with clinicopathological characteristics. (**A**) Differential expression of miR-589-3p in normal lung tissues (*n* = 44) and primary LUAD tissues (*n* = 447) based on TCGA-LUAD miRNA-seq data analyzed using the UALCAN platform. (**B**) miR-589-3p expression stratified by sex in LUAD patients. (**C**) miR-589-3p expression across different clinical stages (Stages I–IV). (**D**) miR-589-3p expression according to lymph node metastasis status (N0–N2). The N3 subgroup was excluded from statistical analysis due to its limited sample size (*n* = 1). Data are presented as the mean ± SD. * *p* < 0.05 was considered to indicate statistical significance.

Analysis of the TCGA-LUAD miRNA sequencing dataset using the UALCAN platform revealed that miR-589-3p expression was significantly higher in primary LUAD tissues than in normal lung tissues (normal, *n* = 44; tumor, *n* = 447; *p* < 0.05; Figure 1A). Stratification by sex demonstrated that miR-589-3p expression was markedly elevated in both male (*n* = 208) and female (*n* = 239) LUAD patients compared with normal controls (*p* < 0.05 for both), whereas no statistically significant difference was observed between male and female tumor samples (Figure 1B). Further subgroup analysis showed that miR-589-3p expression was consistently upregulated across all clinical stages (Stages I–IV) as compared with normal lung tissues (all *p* < 0.05; Figure 1C). In the nodal metastasis subgroup analysis, miR-589-3p expression showed comparable distributions across the N0, N1, and N2 groups, with substantial overlap observed between groups (Figure 1D). Therefore, no definitive association between miR-589-3p expression and nodal status could be concluded from this analysis. Due to the limited sample size in the N3 subgroup (*n* = 1), no statistical analysis was performed for this group. Furthermore, owing to the limitations of the UALCAN platform, formal pairwise statistical comparisons were not available, and these findings should be interpreted with caution.

### 3.2. miR-589-3p Is Overexpressed in LUAD Cell Lines, and Its Inhibition Suppresses Cell Proliferation

**Figure 2:**
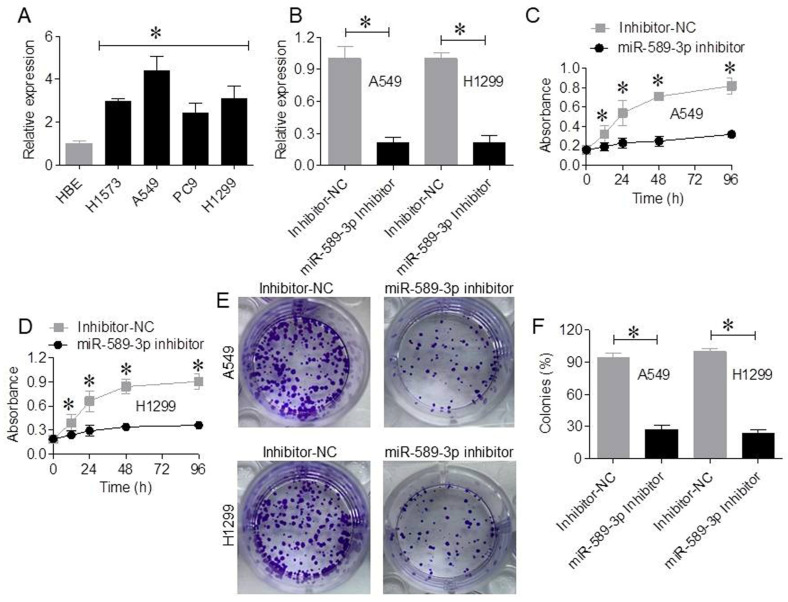
miR-589-3p is overexpressed in LUAD cell lines, and its inhibition suppresses cell proliferation. (**A**) qRT-PCR analysis of miR-589-3p expression in LUAD cell lines (H1975, A549, PC9, and H1299) and normal human bronchial epithelial (HBE) cells. (**B**) Validation of miR-589-3p knockdown efficiency in A549 and H1299 cells following transfection with miR-589-3p inhibitor. (**C**,**D**) CCK-8 assays showing time-dependent effects of miR-589-3p inhibition on the proliferation of A549 and H1299 cells. (**E**,**F**) Representative images and quantitative analysis of colony formation assays in A549 and H1299 cells following miR-589-3p inhibition. Data are expressed as the mean ± SD of three independent experiments. * *p* < 0.05 indicates statistical significance.

qRT-PCR analysis showed that miR-589-3p expression was significantly increased in LUAD cell lines (H1975, A549, PC9, and H1299) compared with normal human bronchial epithelial (HBE) cells (*p* < 0.05), with the highest expression detected in A549 cells (Figure 2A). A549 and H1299 cells were selected for functional assays based on their relatively higher endogenous expression levels of miR-589-3p, which allowed for a more robust evaluation of its biological effects. Transfection with a miR-589-3p inhibitor significantly reduced miR-589-3p expression in both A549 and H1299 cells, as compared with inhibitor-NC (*p* < 0.05; Figure 2B). CCK-8 assays demonstrated that inhibition of miR-589-3p significantly suppressed the proliferation of A549 and H1299 cells in a time-dependent manner as compared with controls (*p* < 0.05; Figure 2C,D). Colony formation assays further showed that miR-589-3p inhibition reduced colony numbers to 27% in A549 cells and 24% in H1299 cells (*p* < 0.05; Figure 2E,F); these values are expressed as percentages relative to the total number of colonies observed in the control group.

### 3.3. WWC2 Is Identified as a Direct Downstream Target of miR-589-3p

**Figure 3:**
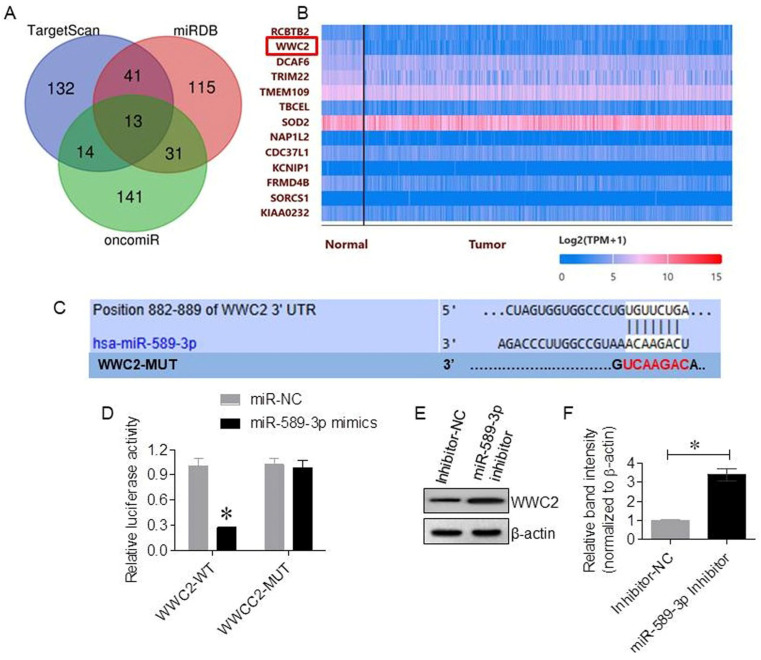
WWC2 is a direct downstream target of miR-589-3p. (**A**) A Venn diagram showing overlapping predicted target genes of miR-589-3p identified using the TargetScan, miRDB, and OncomiR databases. (**B**) Expression levels of WWC2 in normal lung tissues and LUAD tissues based on TCGA-LUAD mRNA-seq data. (**C**) A schematic representation of the predicted binding site between miR-589-3p and the 3′-UTR of WWC2 mRNA, including wild-type and mutant sequences. (**D**) Dual-luciferase reporter assays demonstrating reduced luciferase activity in cells co-transfected with miR-589-3p mimics and WWC2 wild-type reporter but not mutant reporter. (**E**) Western blot analysis showing increased WWC2 protein expression following miR-589-3p inhibition. The uncropped blots are shown in Supplementary Materials. (**F**) Densitometry analysis showing WWC2 protein expression following miR-589-3p inhibition. * *p* < 0.05 was considered to indicate statistical significance.

Bioinformatics analysis using the TargetScan, miRDB, and OncomiR databases was performed to predict potential downstream targets of miR-589-3p. To improve prediction reliability and reduce false-positive results, the top 200 candidate genes from each database were selected, and overlapping targets were identified. A total of 13 common candidate genes were obtained (Figure 3A). Among these, WWC2 was selected for further validation because it was predicted by all three databases and also exhibited significantly reduced expression in LUAD tissues compared with normal lung tissues in a TCGA analysis (Figure 3B), suggesting potential biological relevance. For subsequent mechanistic analyses, A549 cells were used to maintain experimental consistency and minimize inter-cell-line variability, thereby enabling clearer interpretation of the miR-589-3p/WWC2 regulatory relationship. Sequence analysis revealed a conserved binding site for miR-589-3p within the 3′-UTR of WWC2 mRNA (Figure 3C). To validate whether this predicted interaction represents direct binding, dual-luciferase reporter assays were performed. The assays showed that miR-589-3p mimics significantly reduced the luciferase activity of the WWC2 wild-type reporter to approximately 30% of the control level (*p* < 0.05), whereas no significant change was observed in the mutant construct (*p* > 0.05; Figure 3D). This result indicates that miR-589-3p directly binds to the predicted site within the WWC2 3′-UTR in a sequence-specific manner. Inhibition of miR-589-3p in A549 cells resulted in a considerable increase in WWC2 protein expression compared with that in control cells (Figure 3E,F), further supporting a negative regulatory relationship between miR-589-3p and WWC2.

### 3.4. WWC2 Is Downregulated in LUAD Tissues and Exhibits Tumor-Suppressive Effects In Vitro

**Figure 4:**
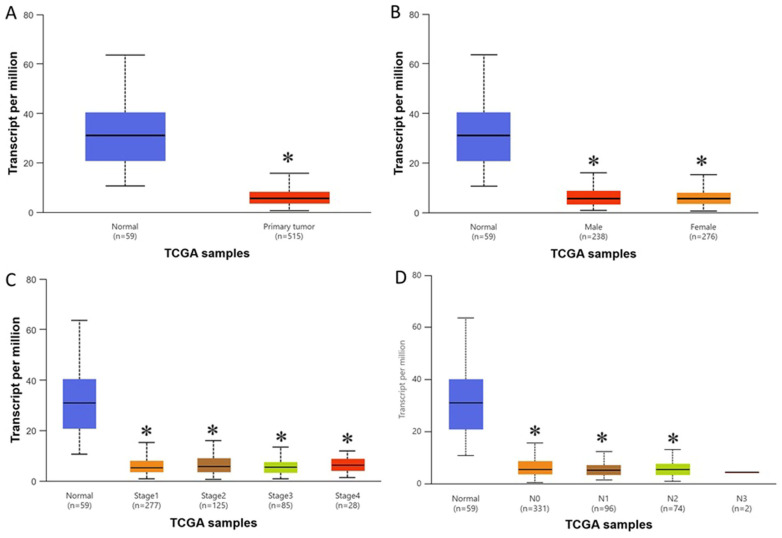
WWC2 is downregulated in LUAD tissues. (**A**) WWC2 expression in normal lung tissues (*n* = 59) and LUAD tissues (*n* = 515) based on TCGA-LUAD datasets. (**B**) WWC2 expression stratified by sex. (**C**) WWC2 expression across different tumor stages. (**D**) WWC2 expression according to lymph node metastasis status. Data were obtained using the UALCAN platform. * *p* < 0.05 indicates statistical significance.

**Figure 5:**
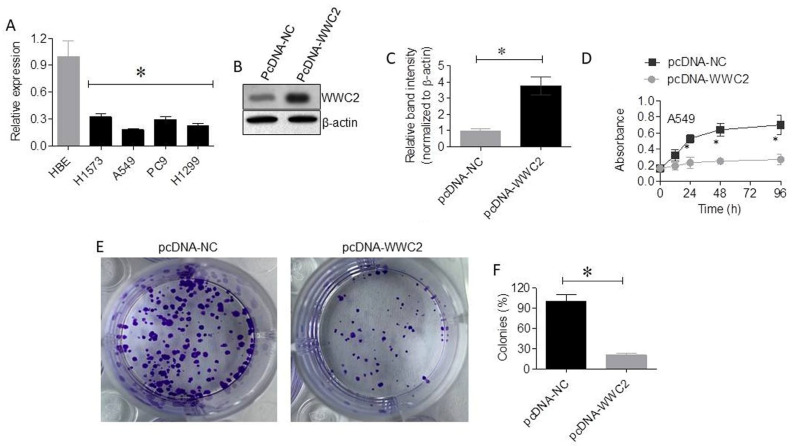
WWC2 overexpression inhibits LUAD cell proliferation and colony formation. (**A**) qRT-PCR analysis of WWC2 expression in LUAD cell lines and HBE cells. The uncropped blots are shown in Supplementary Materials. (**B**) Western blot validation of WWC2 overexpression in A549 cells. (**C**) Densitometry analysis showing WWC2 overexpression in A549 cells. (**D**) CCK-8 assays showing the effect of WWC2 overexpression on A549 cell proliferation over time. (**E**,**F**) Representative images and quantitative analysis of colony formation assays following WWC2 overexpression. Data are presented as the mean ± SD from three independent experiments. * *p* < 0.05 was considered to indicate statistical significance.

TCGA-LUAD analysis using UALCAN demonstrated that WWC2 expression was significantly lower in LUAD tissues than in normal lung tissues (normal, *n* = 59; tumor, *n* = 515; *p* < 0.05; Figure 4A). This downregulation was consistently observed across sexes (*p* < 0.05; Figure 4B), tumor stages (*p* < 0.01; Figure 4C), and nodal metastasis statuses (*p* < 0.05; Figure 4D). qRT-PCR analysis confirmed that WWC2 expression was significantly reduced in LUAD cell lines compared with HBE cells (*p* < 0.05; Figure 5A). Overexpression of WWC2 in A549 cells led to a considerable increase in its protein levels (Figure 5B,C) and significantly suppressed cell proliferation, time-dependently reducing cell viability (*p* < 0.05; Figure 5D). Colony formation assays showed that WWC2 overexpression in A549 cells reduced colony numbers by around 79% relative to those observed for the control (*p* < 0.05; Figure 5E,F).

### 3.5. WWC2 Mediates the Proliferative Effects of miR-589-3p in LUAD Cells

**Figure 6:**
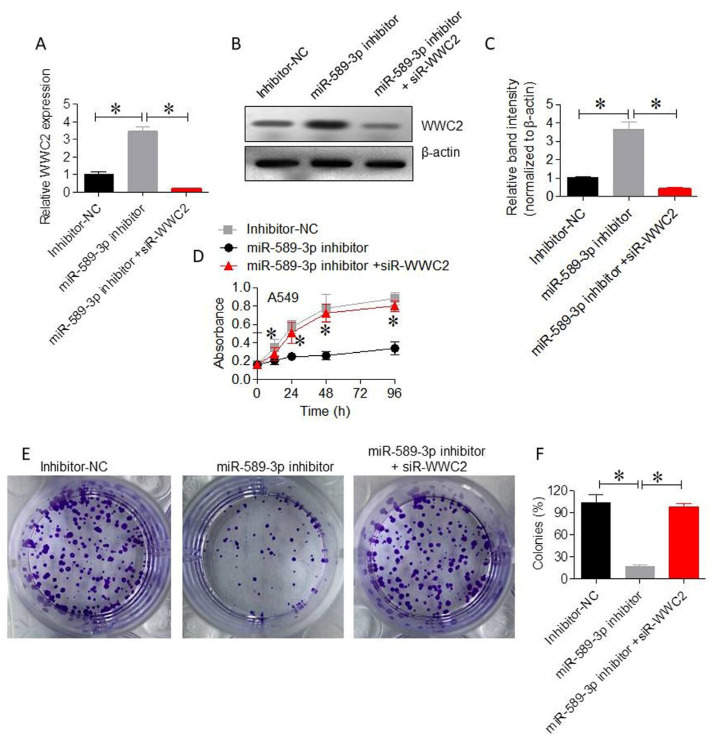
WWC2 mediates the proliferative effects of miR-589-3p in LUAD cells. (**A**) qRT-PCR showing the expression of WWC2 in A549 cells transfected with miR-589-3p inhibitor alone or in combination with si-WWC2. (**B**) Western blot analysis of WWC2 protein expression in A549 cells transfected with miR-589-3p inhibitor alone or in combination with si-WWC2. The uncropped blots are shown in Supplementary Materials. (**C**) Densitometry analysis showing WWC2 protein expression in A549 cells transfected with miR-589-3p inhibitor alone or in combination with si-WWC2. (**D**) CCK-8 assays showing restoration of cell viability following WWC2 knockdown in miR-589-3p-inhibited cells. (**E**,**F**) Representative images and quantitative analysis of colony formation assays demonstrating the reversal of growth inhibition by WWC2 silencing. Data are shown as the mean ± SD. * *p* < 0.05 indicates statistical significance.

qRT-PCR and Western blot analysis demonstrated that miR-589-3p inhibition in A549 cells led to a considerable increase in WWC2 mRNA and protein expression, which was effectively reversed by co-transfection with si-WWC2 (Figure 6A–C). CCK-8 assays showed that WWC2 knockdown restored cell viability in miR-589-3p-inhibited A549 cells (*p* < 0.01; Figure 6D). Similarly, colony formation assays revealed that WWC2 silencing increased colony numbers, reversing the inhibitory effect of miR-589-3p suppression (*p* < 0.01; Figure 6E,F).

### 3.6. miR-589-3p Regulates LUAD Cell Apoptosis Through WWC2

**Figure 7:**
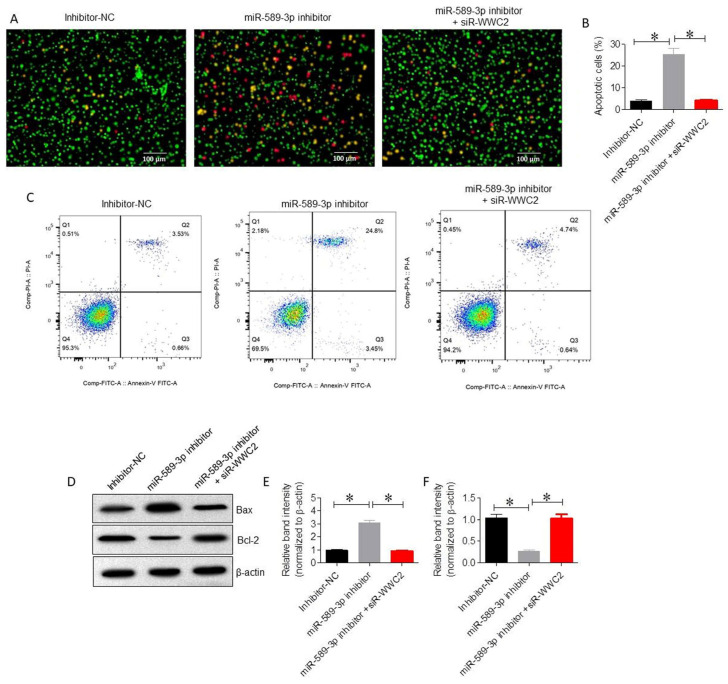
miR-589-3p regulates LUAD cell apoptosis through WWC2. (**A**) Representative fluorescence images of AO/EB staining showing apoptotic cells following miR-589-3p inhibition and co-transfection with si-WWC2. Live cells appear green, early apoptotic cells show bright green nuclei, late apoptotic cells appear orange/red, and necrotic cells are red. (**B**) Quantification of apoptotic cells detected via AO/EB staining. (**C**) Flow cytometric analysis of apoptosis using Annexin V-FITC/PI staining, with quantification of early and late apoptotic cells. Q4 represents viable cells, Q3 early apoptosis, Q2 late apop-tosis, and Q1 necrosis. (**D**) Western blot analysis of apoptosis-related proteins Bax and Bcl-2 under indicated transfection conditions. The uncropped blots are shown in Supplementary Materials. (**E**) Densitometry analysis showing Bax expression and (**F**) Bcl-2 expression in A549 cells under indicated conditions. Data represent the mean ± SD of three independent experiments. * *p* < 0.05 was considered to indicate statistical significance.

To further investigate whether miR-589-3p influences apoptotic processes in LUAD cells and to elucidate the underlying molecular mechanisms, AO/EB staining and Western blot analysis of apoptosis-related proteins (Bax and Bcl-2) were performed. AO/EB staining demonstrated that miR-589-3p inhibition significantly increased the proportion of apoptotic A549 cells, while WWC2 knockdown reduced apoptosis (*p* < 0.01; Figure 7A,B). Flow cytometric analysis further confirmed that total apoptosis increased from 4.19% to 28.25% following miR-589-3p inhibition but was reduced to 5.4% upon si-WWC2 co-transfection (*p* < 0.01; Figure 7C). Western blot analysis showed that miR-589-3p inhibition increased Bax expression and decreased Bcl-2, while WWC2 silencing reversed these effects in A549 cells (Figure 7D–F).

### 3.7. miR-589-3p Promotes LUAD Cell Invasion Via WWC2 Suppression

**Figure 8:**
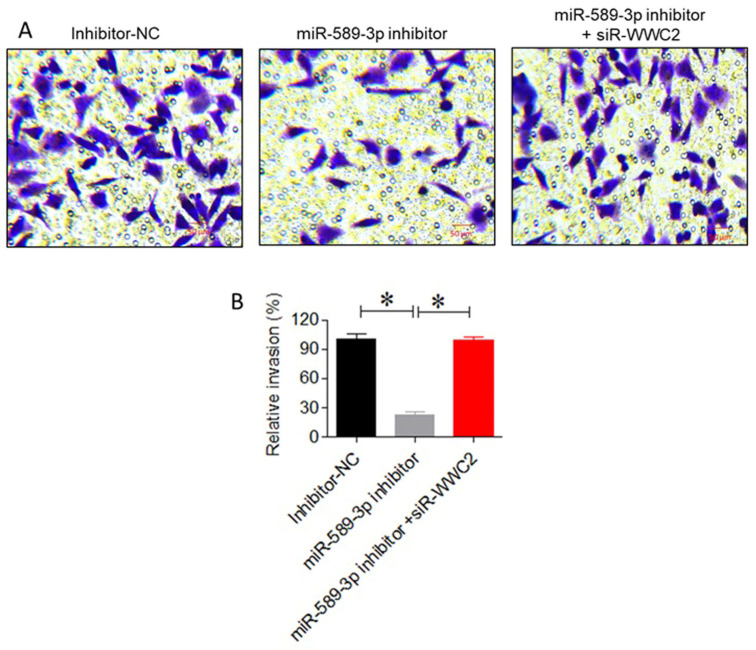
miR-589-3p promotes LUAD cell invasion via suppression of WWC2. (**A**) Representative images of Transwell invasion assays in A549 cells following miR-589-3p inhibition and co-transfection with si-WWC2. Blue color indicate the crystal violet stained cells that have invaded. (**B**) Quantitative analysis of invaded cells showing reduced invasion upon miR-589-3p inhibition and restoration following WWC2 knockdown. Data are expressed as the mean ± SD. * *p* < 0.05 indicates statistical significance.

Transwell invasion assays demonstrated that miR-589-3p inhibition reduced the number of invasive cells to 22.6% that observed for the control (*p* < 0.05; Figure 8A,B). Co-transfection with si-WWC2 restored the invasive capacity of A549 cells, confirming that WWC2 mediates the pro-invasive effects of miR-589-3p (*p* < 0.01).

## 4. Discussion

In this study, we demonstrated that miR-589-3p is significantly upregulated in lung adenocarcinoma and plays a promotive role in LUAD cell proliferation and invasion while suppressing apoptosis. Through integrated bioinformatics analysis and experimental validation, we identified WWC2 as a direct downstream target of miR-589-3p and showed that restoration of WWC2 expression reverses the oncogenic effects induced by miR-589-3p. These findings collectively establish a functional miR-589-3p/WWC2 regulatory axis that contributes to LUAD progression. Analyses of TCGA-LUAD datasets revealed that miR-589-3p expression was significantly elevated in LUAD tissues compared with normal lung tissues and was associated with the tumor stage but not definitively associated with nodal metastasis, as substantial overlap in expression distributions was observed among the nodal subgroups. Due to the limitations of the UALCAN platform, formal pairwise statistical comparisons and trend analyses were not performed, and these associations should be interpreted with caution. This limitation reduces the strength of conclusions regarding clinicopathological correlations, particularly for nodal status. Our observations suggest that miR-589-3p upregulation may be involved in LUAD progression rather than merely reflecting tumor presence. The consistency between TCGA data and LUAD cell line expression further supports a biologically relevant role for miR-589-3p in lung adenocarcinoma. Dysregulated miRNAs are increasingly recognized as key modulators of tumor aggressiveness, acting as fine-tuners of oncogenic signaling pathways rather than primary drivers [9,10,27]. Functionally, inhibition of miR-589-3p significantly suppressed LUAD cells’ proliferation and clonogenic capacity while promoting their apoptotic cell death and reducing their invasive potential. However, these functional findings were primarily derived from in vitro experiments, and the absence of in vivo validation limits the translational strength of these conclusions. These findings are consistent with previous reports demonstrating that miR-589 regulates proliferation, migration, and apoptosis in various malignancies, although its functional role appears to be highly context-dependent [13,14,15,16,17,18,19,20]. In breast cancer, miR-589-3p acts as a tumor suppressor by inhibiting Akt signaling via IGF1R repression [17], whereas in gastric cancer and pancreatic cancer, miR-589 promotes tumor aggressiveness through oncogenic feedback loops [19,20]. Our results expand the oncogenic spectrum of miR-589-3p and provide the first direct evidence of its functional significance in lung adenocarcinoma. A previous study [12] reported that miR-589-5p is epigenetically silenced in NSCLC through promoter hypermethylation, thereby contributing to tumor progression. However, in the present study, miR-589-3p was found to be upregulated in LUAD tissues and cell lines. This apparent difference may be attributed to the fact that miR-589-5p and miR-589-3p are derived from the same precursor but can exhibit distinct expression patterns and biological functions due to differential strand selection and processing. Variations in tumor subtype and experimental context may also contribute to these differences. Given the limited available evidence, further studies are required to clarify the specific roles of miR-589-5p and miR-589-3p in lung cancer. WWC2 emerged as a critical mediator of miR-589-3p-driven effects in LUAD. Consistent with previous studies, we observed that WWC2 expression was significantly downregulated in LUAD tissues and cell lines [21,25]. Overexpression of WWC2 suppressed LUAD cell proliferation and colony formation, supporting its tumor-suppressive role. Prior investigations have demonstrated that WWC2 inhibits invasion and metastasis in hepatocellular carcinoma and colorectal cancer, partly through modulation of Hippo signaling [22,23,24]. Our findings align with these reports and further highlight the importance of WWC2 in restraining malignant phenotypes across different cancer types. Importantly, we confirmed a direct regulatory interaction between miR-589-3p and WWC2 through dual-luciferase reporter assays and rescue experiments. Suppressing miR-589-3p increased WWC2 expression and inhibited LUAD cell proliferation and invasion, whereas silencing WWC2 reversed these effects. These results provide strong mechanistic evidence that miR-589-3p promotes LUAD progression, at least in part, by repressing WWC2 expression. However, the mechanistic experiments were primarily conducted in a single LUAD cell line (A549), which may limit the generalizability of these findings across heterogeneous LUAD models. Such miRNA–mRNA regulatory axes are increasingly recognized as central components of cancer-associated signaling networks [11,12,28]. Apoptosis assays further revealed that miR-589-3p inhibition significantly increased apoptotic cell populations, accompanied by upregulation of the pro-apoptotic protein Bax and downregulation of the anti-apoptotic protein Bcl-2. These changes were reversed by WWC2 knockdown, indicating that WWC2 mediates the pro-apoptotic effects observed following miR-589-3p inhibition. Dysregulation of apoptosis is a hallmark of lung cancer, and miRNAs are known to modulate apoptotic signaling by targeting key regulatory molecules [24,29]. Our findings suggest that the miR-589-3p/WWC2 axis is associated with the modulation of apoptosis-related proteins, although the precise regulatory mechanism remains to be fully elucidated. The absence of complementary migration assays and mimic-based validation of endogenous WWC2 expression further limits the mechanistic completeness of this study.

Taken together, while our findings provide strong preliminary evidence supporting the oncogenic role of the miR-589-3p/WWC2 axis in LUAD, the above limitations indicate that the conclusions should be interpreted with appropriate caution, and further in vivo and multi-model validation studies are required to strengthen their translational relevance.

## 5. Conclusions

In conclusion, this study demonstrated that miR-589-3p is aberrantly upregulated in lung adenocarcinoma and plays a promotive role in tumor cell proliferation and invasion while suppressing apoptosis. Through integrated bioinformatics analysis and in vitro functional assays, we identified WWC2 as a direct downstream target of miR-589-3p and confirmed that repression of WWC2 mediates the oncogenic effects of miR-589-3p in LUAD cells. Restoration of WWC2 expression significantly attenuated the malignant phenotypes induced by miR-589-3p, highlighting the functional importance of the miR-589-3p/WWC2 regulatory axis in lung adenocarcinoma progression. Although further in vivo and clinical validation is required, these findings provide new insight into miRNA-mediated regulatory mechanisms in LUAD and establish a foundation for future studies exploring the biological and translational relevance of the miR-589-3p/WWC2 axis.

## Data Availability

The original contributions presented in this study are included in the article/supplementary material. Further inquiries can be directed to the corresponding author.

## Supplementary Materials

The following supporting information can be downloaded at: https://www.mdpi.com/article/10.3390/cancers18091349/s1, Table S1: The primers used for qRT-PCR analysis in this study; Supplementary File: Uncropped western blots.
