# Supplementary material for: Upregulation of miR-589-3p Contributes to Lung Adenocarcinoma Progression Through Inhibition of WWC2
Source: Cancers (Basel). 2026 Apr 23;18(9):1349. doi: 10.3390/cancers18091349 (PMC13162627; doi:10.3390/cancers18091349)
Supplement: Supplementary file 1 [file cancers-18-01349-s001.zip › cancers-4213827-supplementary/cancers-4213827-supplementary.pdf]

Supplementary Table S1. Primers used for qRT-PCR analysis in this study.

Target	Primer	Sequence (5′→3′)
**miR-589-3p**	Forward	AACAAATGCCGGTTCCCAGA
	Reverse	TGTCGTGGAGTCGGCAATTG
**U6 snRNA**	Forward	TGCGGTGGGTGTCATCAAA
	Reverse	AACGCTTCACGAATTTGGT
**WWC2**	Forward	CTGATGAGTCTGTGGCTGGAGA
	Reverse	CGGTCTCTACAATGGCTACATCC
**GAPDH**	Forward	GGCAAATTCCATGGCACCGT
	Reverse	GCATCGCCCCACTTGATTTT
