# Supplementary figures and images for: Upregulation of miR-589-3p Contributes to Lung Adenocarcinoma Progression Through Inhibition of WWC2
Source: Cancers (Basel). 2026 Apr 23;18(9):1349. doi: 10.3390/cancers18091349 (PMC13162627; doi:10.3390/cancers18091349)

**Figure 3E**

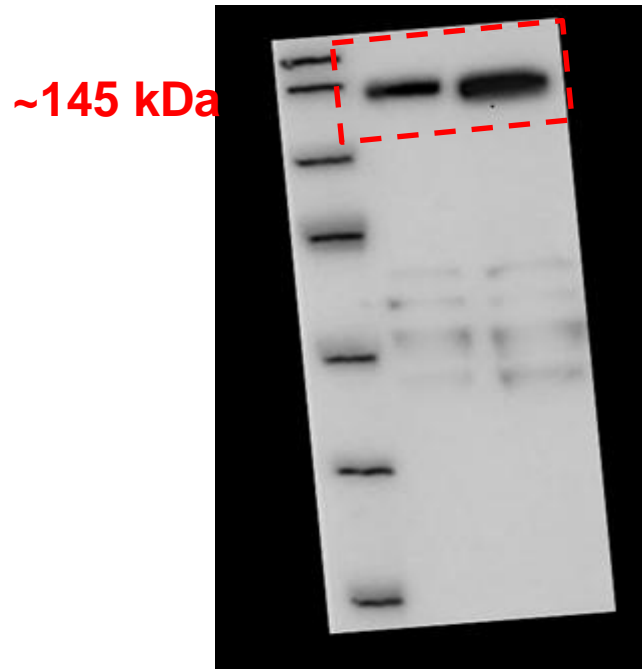

**WWC2**

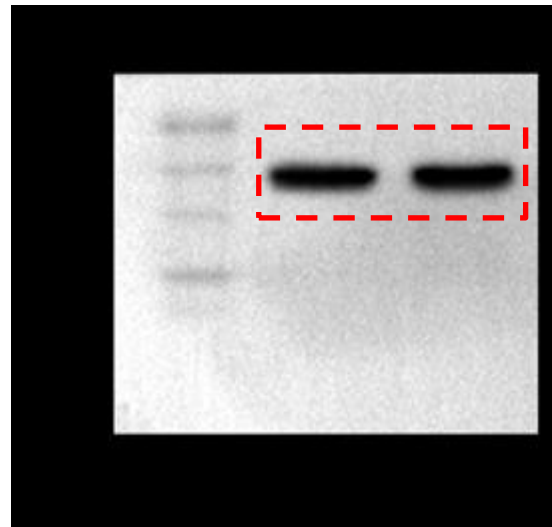

**~ 42 kDa**

**Actin**

**Figure 5B**

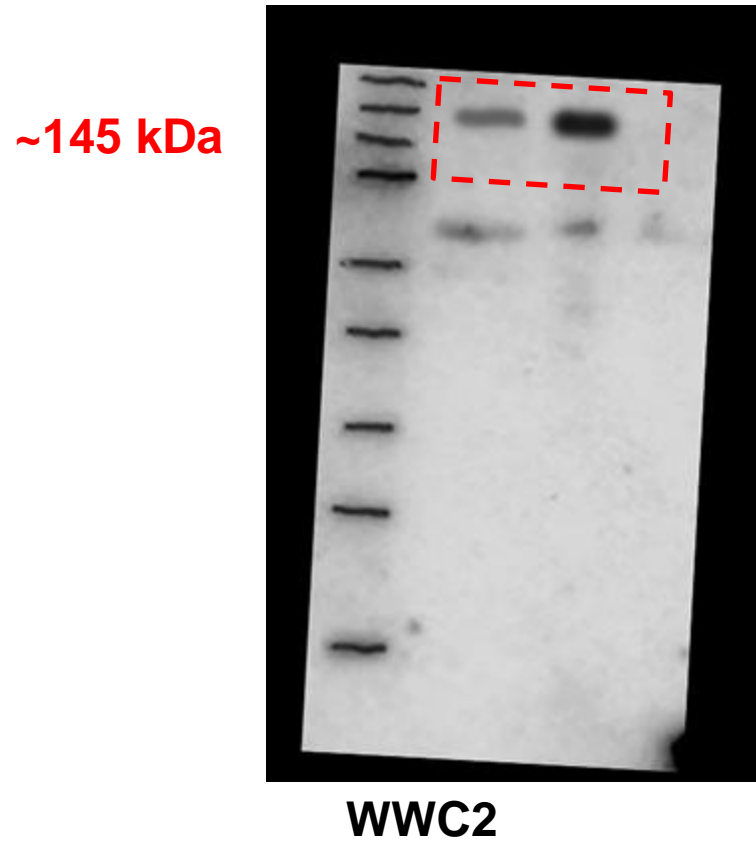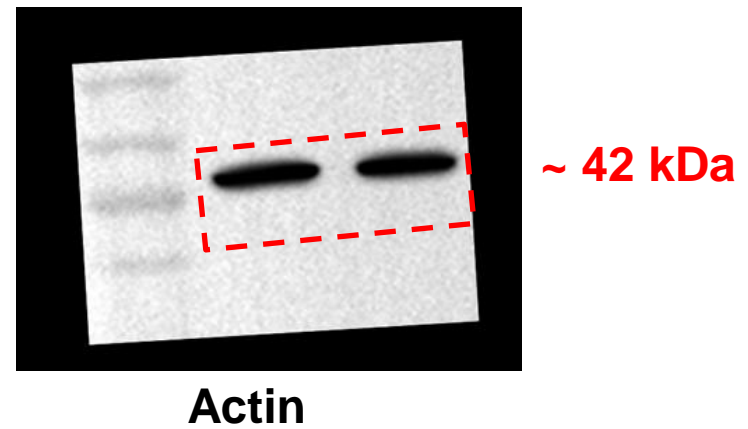

**Figure 5B**

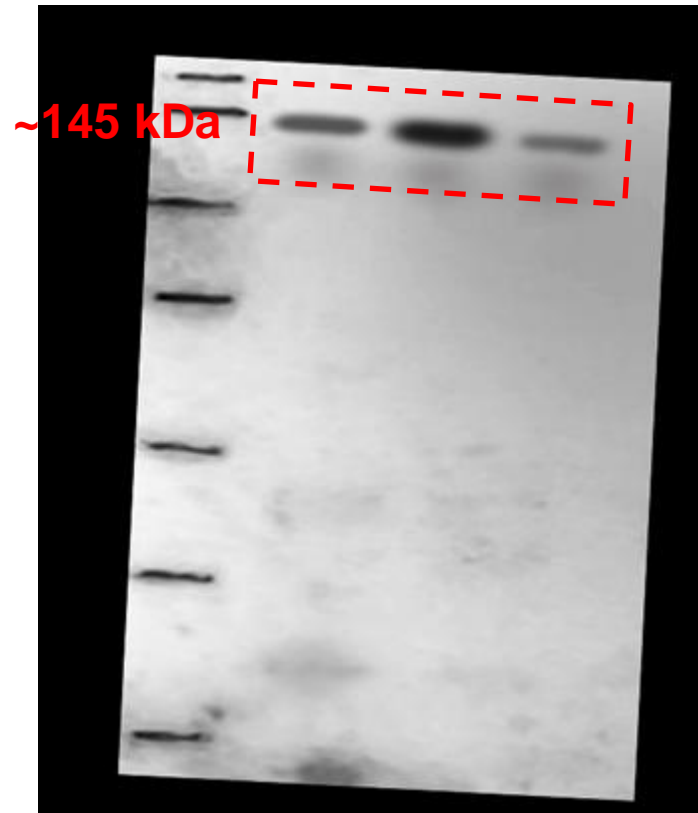

**WWC2**

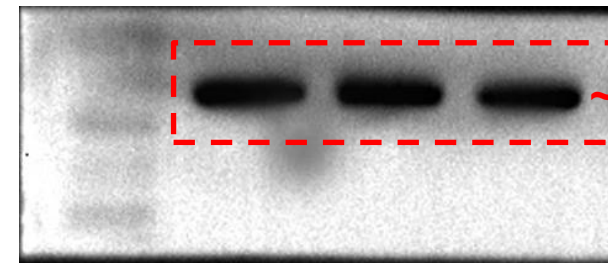

**Actin**

**Figure 7C**

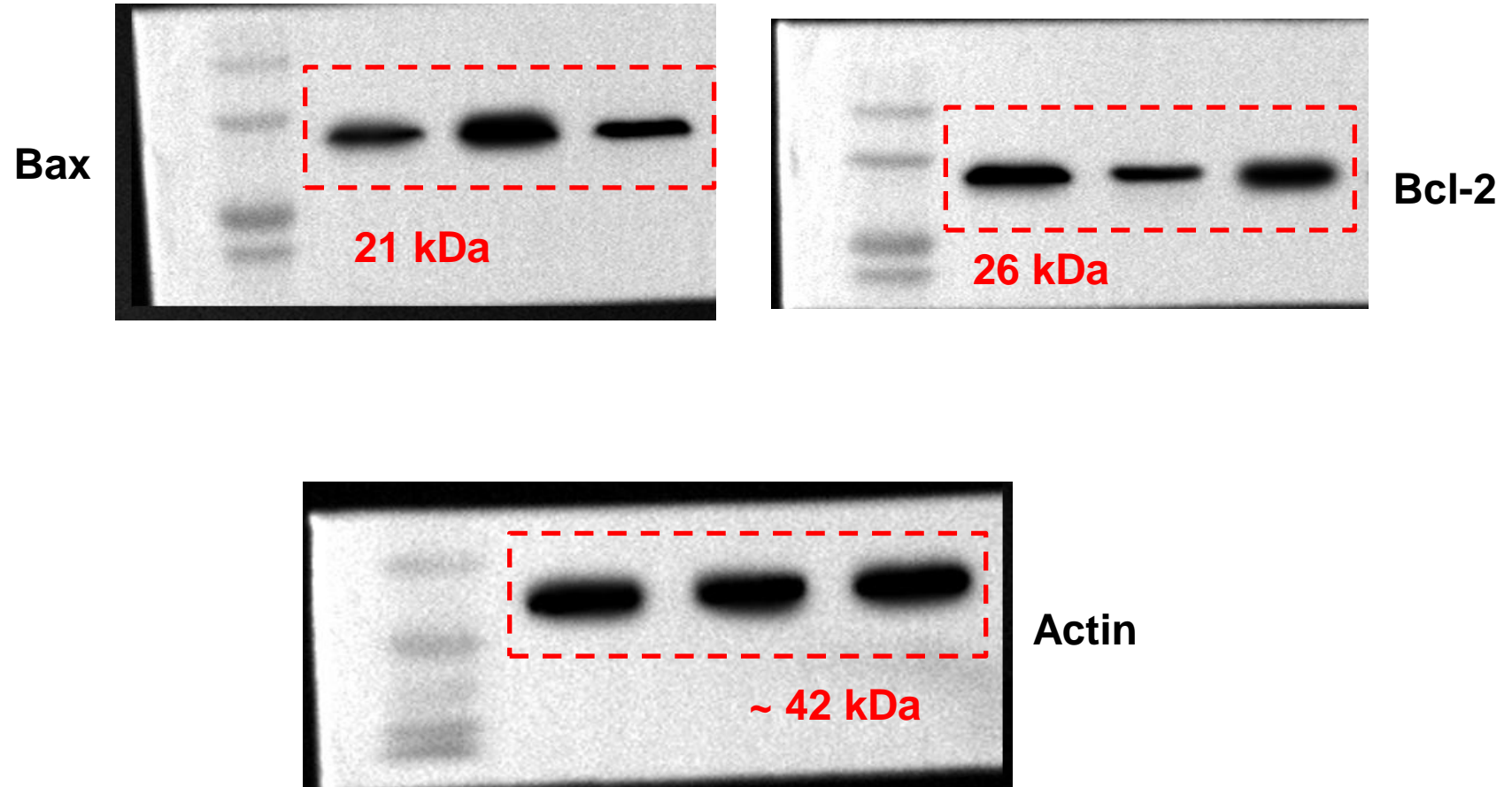

Supplement: Supplementary file 1 [file cancers-18-01349-s001.zip › cancers-4213827-supplementary/Uncropped western blots.pdf]
